# Resource availability governs polyhydroxyalkanoate (PHA) accumulation and diversity of methanotrophic enrichments from wetlands

**DOI:** 10.3389/fbioe.2023.1210392

**Published:** 2023-07-31

**Authors:** Yujin Kim, Zachary Flinkstrom, Pieter Candry, Mari-Karoliina H. Winkler, Jaewook Myung

**Affiliations:** ^1^ Department of Civil and Environmental Engineering, Korea Advanced Institute of Science and Technology (KAIST), Daejeon, Republic of Korea; ^2^ Department of Civil and Environmental Engineering, University of Washington, Seattle, WA, United States

**Keywords:** methanotrophs, polyhydroxyalkanoate (PHA), wetlands, resource availability, enrichments, bioplastics

## Abstract

Aquatic environments account for half of global CH_4_ emissions, with freshwater wetlands being the most significant contributors. These CH_4_ fluxes can be partially offset by aerobic CH_4_ oxidation driven by methanotrophs. Additionally, some methanotrophs can convert CH_4_ into polyhydroxyalkanoate (PHA), an energy storage molecule as well as a promising bioplastic polymer. In this study, we investigate how PHA-accumulating methanotrophic communities enriched from wetlands were shaped by varying resource availability (i.e., C and N concentrations) at a fixed C/N ratio. Cell yields, PHA accumulation, and community composition were evaluated in high (20% CH_4_ and 10 mM NH_4_^+^) and low resource (0.2% CH_4_ and 0.1 mM NH_4_^+^) conditions simulating engineered and environmental settings, respectively. High resource availability decreased C-based cell yields, while N-based cell yields remained stable, suggesting nutrient exchange patterns differed between methanotrophic communities at different resource concentrations. PHA accumulation was only observed in high resource enrichments, producing approximately 12.6% ± 2.4% (m/m) PHA, while PHA in low resource enrichments remained below detection. High resource enrichments were dominated by *Methylocystis* methanotrophs, while low resource enrichments remained significantly more diverse and contained only a minor population of methanotrophs. This study demonstrates that resource concentration shapes PHA-accumulating methanotrophic communities. Together, this provides useful information to leverage such communities in engineering settings as well as to begin understanding their role in the environment.

## 1 Introduction

Despite the groundbreaking relevance of plastics as synthetic materials, they also have become a global threat to the environment due to their durability and resistance to degradation under ambient conditions (MacLeod et al., 2021). An estimated 12,000 Mt of plastic waste will be deposited into landfills or the environment by 2050 (Chamas et al., 2020). To address plastic waste issues, polyhydroxyalkanoates (PHAs)—microbially produced polyesters with applications in packaging and medical industries (Tan et al., 2014)—have been proposed as alternative bioplastics due to their high biodegradability (Choe et al., 2022) and biocompatibility (Koller, 2018).

Some microorganisms can produce and accumulate PHA-granules in their cell biomass as a strategy for energy storage to overcome periods where nutrients (e.g., nitrogen, phosphorus) are limited but carbon is still readily available (Pieja et al., 2017). Short-chain organic acids (e.g., acetate, propionate) have been widely explored as carbon substrates for PHA production (Albuquerque et al., 2007; Johnson et al., 2010). More recently, methanotrophic PHA accumulators have been explored as a powerful tool to upgrade low-value biogas (CH_4_/CO_2_) streams to bioplastics. These methanotrophic PHA-accumulators are type II methanotrophs (i.e., using the serine cycle for biomass synthesis (Nguyen et al., 2021)) capable of converting CH_4_ gas into PHA granules (Rostkowski et al., 2013). Typical type II methanotrophs are *Methylocystis*, *Methylosinus*, *Methylocella*, *Methylocapsa*, and *Methyloferula*. While these organisms are widely distributed, most studies enriching PHA-accumulating methanotrophic communities have focused on activated sludge from wastewater treatment plants, leaving their presence and role in natural settings underexplored (Pieja et al., 2011; Strong et al., 2016; Pérez et al., 2019).

While PHA-accumulating methanotrophic microorganisms have been excessively studied in engineered settings, research on their relevance to the natural environment remains sparse. However, in the face of defining the origins and sinks of climate change-related greenhouse budgets, there is an urgent need to study their role more holistically in natural systems. In specific, freshwater wetlands contribute approximately 20%–30% of global CH_4_ emissions (Rosentreter et al., 2021). Given the large global warming potential of CH_4_, about 30 CO_2_ equivalents over a 100-year time horizon, even small quantities of CH_4_ can largely affect the carbon footprint. Thus, a reduction of CH_4_ to CO_2_ through methanotrophs can significantly lower the emission factor from wetlands. In periodically flooded wetlands, alternating anaerobic and aerobic conditions promote the production and consumption of CH_4_, respectively. This pattern is especially pronounced in the thin oxic layer near the sediment-water interface where methanotrophs oxidize CH_4_ to CO_2_, counteracting some of these CH_4_ fluxes (Chowdhury and Dick, 2013). Moreover, aerobic CH_4_ oxidation has been suggested to be the key process that offsets the CH_4_ fluxes generated from saturated wetland soils (Chowdhury and Dick, 2013). Despite the critical role of methanotrophs in wetlands, their capacity for PHA accumulation remains untested.

Most studies have investigated PHA accumulation in methanotrophs under high (>20%) CH_4_ concentrations (Pieja et al., 2012; Myung et al., 2015; López et al., 2018). Under these conditions, the C/N ratio was found to affect PHA production and methanotrophic growth yield (Rostkowski et al., 2013; Zaldívar Carrillo et al., 2018). However, the impact of resource availability (i.e., concentration) on PHA-accumulating methanotrophs remains uncertain. One study investigated kinetic properties and substrate affinities of methanotrophic cultures enriched at low CH_4_ concentrations (200 ppm) (Dunfield et al., 1999). While this community harbored type II methanotrophs, the potential for PHA accumulation was not evaluated.

In this study, the impact of carbon and nitrogen resource availability on methanotrophic cultures enriched from freshwater wetland sediments was investigated. Communities were enriched under high and low resource (i.e., CH_4_, NH_4_^+^) conditions, to represent engineered and natural systems, respectively. Under these conditions, cell growth and substrate consumption were evaluated in batch cultures, PHA accumulation was tested to examine the potential implications for biorefineries, and differences in microbial community composition were evaluated.

## 2 Materials and methods

### 2.1 Methanotrophic culture enrichment

Three different soil cores were obtained from a seasonally flooded urban freshwater wetland connected to Lake Washington in Seattle, WA, United States (Coordinates: 47.642196° N, −122.296236° W) using a soil core sampler (AMS, Inc., American Falls, ID). The cores were transported to the laboratory on ice for same-day processing. The cores were not mixed, and 1 mL of soil from the surface of each core was inoculated into 50 mL of ammonium mineral salts (AMS) medium. AMS medium was chosen based on its ability to enrich PHA-accumulating methanotrophs (Myung et al., 2015). The basal AMS medium contained (per 1,000 mL): 1 g of MgSO_4_.7H_2_O, 0.2 g of CaCl_2_.2H_2_O, 0.1 mL of a 3.8% (w/v) Fe(III)-EDTA solution, 0.5 mL of a 0.1% (w/v) Na_2_MoO_4_.2H_2_O solution, and 1 mL of a trace element solution. The trace element solution contained (per L): 0.5 g of FeSO_4_.7H_2_O, 0.4 g of ZnSO_4_.7H_2_O, 0.02 g of MnCl_2_.7H_2_O, 0.05 g of CoCl_2_.6H_2_O, 0.01 g of NiCl_2_.6H_2_O, 0.015 g of H_3_BO_3_ and 0.25 g of EDTA. After autoclaving, 10 mL of 0.4 M of phosphate buffer solution adjusted to pH 6.8 with KH_2_PO_4_ and Na_2_HPO_4_.7H_2_O, 1 mL of a 10-fold concentrated filter-sterilized vitamin stock (which contained 20 mg of biotin, 20 mg of folic acid, 50 mg of thiamine HCl, 50 mg of Ca pantothenate, 1 mg of vitamin B12, 50 mg of riboflavin, and 50 mg of nicotinamide per L), and 1 mL of a 10 mM CuCl_2_.2H_2_O solution were injected into the 1 L-medium. Serum bottles (160 mL total volume, Wheaton, Millville, NJ) were filled with 50 mL of medium, capped with butyl-rubber stoppers, and crimp-sealed under atmospheric pressure.

In this study, we investigated two conditions: 1) high resource enrichments with 20% CH_4_ (99.999%, Linde, Danbury, CT) and 10 mM NH_4_^+^ (0.53 g NH_4_Cl·L^-1^), and 2) low resource enrichments with 0.2% CH_4_ and 0.1 mM NH_4_^+^ (0.0053 g of NH_4_Cl·L^-1^). Bottles were agitated horizontally in a shaking incubator at 150 rpm controlled at a temperature of 30 ^°^C. Communities were enriched in batch cycles, transferring 10% (v/v) after 14 days (first transfer) or every 7 days (every subsequent transfer).

### 2.2 PHA-accumulation incubations

All PHA accumulation cycles were performed with fully grown cultures from routine enrichment described above. Specifically, 40 mL of culture was centrifuged at 4,000 rpm for 15 min. The supernatant was removed, and the biomass pellet was resuspended in 10 mL of fresh nitrogen-depleted AMS medium, which was then injected into serum bottles with 40 mL of nitrogen-depleted AMS medium for a final liquid volume of 50 mL. The headspace was replaced with either 20% or 0.2% of pure CH_4_ gas (99.999%, Linde, Danbury, CT) for high and low resource conditions, respectively. All bottles were agitated horizontally in a temperature-controlled (30 °C) shaking incubator at 150 rpm for 5 days.

### 2.3 Analytical methods

The gas phase of the enrichment cultures was analyzed by gas chromatography (SRI 8610C, SRI Instruments, Torrance, CA) with a 1.83 m Haysep D column and a thermal conductivity detector (TCD) to measure the CH_4_ consumption during the growth cycle. N_2_ carrier gas was continuously injected at 17 psi, and the oven and detector were set at 70 °C and 100 °C, respectively. NH_4_^+^, NO_2_^−^, and TON (NO_3_^−^ + NO_2_^−^) concentrations in the liquid phase were monitored daily with a Gallery™ Discrete Analyzer (Thermo Fisher, United States ) using standard methods (EPA Methods 350.1, 354.1, and 353.1 respectively). Flow cytometry (Guava easyCyte™, Luminex, Austin, TX) was used to measure the cell concentrations during the growth cycle. Samples were diluted 100-fold (high resource) or 10-fold (low resource) in PBS buffer. 1 mL of diluted cell suspension was stained with 10 µL of 30-fold diluted SYBR Green stain (100,00x concentrate, Thermo Fischer) in TE buffer. The stained samples were incubated for 30 min at 37 °C under dark conditions and then analyzed on the flow cytometer.

### 2.4 Microscopic visualization and quantification of PHA granules

PHA granules were stained with Nile Blue A and visualized with epifluorescence microscopy (Lazic et al., 2022). Cells collected after a PHA accumulation cycle were diluted and stained with 10 µL of 30-fold diluted SYBR Green solution and 10 µL Nile Blue A solution (0.05% (w/v) Nile Blue A in ethanol) per milliliter. Stained samples were incubated at 30°C for 30 min. Prior to imaging, stained cells were filtered onto a 0.2 µm Isopore membrane (Cat No. GTBP02500, Millipore, Burlington, MA, United States ) and were imaged on a glass slide with coverslip. An epifluorescent microscope (Axioskop ×2, Zeiss, Germany) equipped with a mercury-arc lamp and ×63Plan-APOCHROMAT objective was used to visualize the cells. Nile Blue stained PHA granules were visualized with a Cy3 filter set (513–556 nm excitation, 570–613 nm emission), Nile Blue stained membranes were visualized with a Cy5 filter set (604–644 nm excitation, 672–712 nm emission), and SYBR Green stained DNA was visualized with a FITC filter set (467–498 nm excitation, 513–556 emission).

Quantitative image analysis was performed using Fiji ImageJ (Schindelin et al., 2012). Images from each fluorescence channel were thresholded using a Bernsen Auto Local Threshold before input into the Analyze Particles plugin. PHA content per cell was calculated using the fraction of membrane area occupied by PHA particles. The percent area was converted to a percent volume by assuming a volume equal to that of a sphere with the measured cross-sectional area. Finally, the percent volume was converted to a percent mass using the 1.099 PHA to biomass density ratio described by (Lazic et al., 2022). To calculate the percentage of cells harboring PHA, the number of cells with detectable PHA signal was divided by the image cell count based on SYBR Green DNA staining.

### 2.5 PHA content measurement

PHA content was quantitatively measured by gas chromatography (GC) following the protocol described in (Myung et al., 2015). After PHA accumulation, the liquid phase was centrifuged at 4,000 rpm for 15 min and the biomass in the pellet was freeze-dried, weighed, and transferred to a 12-mL glass vial. Each sample was amended with 2 mL of chloroform and 2 mL of acidified methanol containing sulfuric acid (3%, vol/vol) and benzoic acid (0.25 mg/mL methanol). All vials were sealed tight with Teflon-lined plastic caps, shaken, and heated at 100°C for 3.5 h. After cooling to room temperature, 1 mL of deionized water was added to create an aqueous phase separated from the chloroform organic phase. The reaction cocktail was vortex mixed for 30 s and then allowed to phase separate. The organic phase was sampled by syringe and analyzed using a GC (Agilent, 8,890) equipped with an HP-5 column (Agilent, 30 m × 0.320 mm × 0.25 µm) and a flame ionization detector (FID). The initial oven temperature was 50 °C (1 min) which was then increased at a rate of 10°C ·min^-1^–160°C, held for 4 min, increased at the same rate to 200°C, held for 4 min, and increased at the same rate again to 275°C, where it was held for 6 min.

### 2.6 Microbial community analysis

Amplicon sequencing of the 16S rRNA gene was conducted to investigate the microbial community composition of each sample from the original wetland soil, and the high and low resource enrichments. DNA was extracted using the DNeasy PowerSoil Pro kit (Qiagen, Hilden, Germany) according to the manufacturer’s protocol. The concentration of DNA was quantified using the Qubit™ dsDNA High Sensitivity Assay (Thermo Fisher Scientific, Waltham, MA, United States ) and normalized with nuclease-free water to 2.5 ng/μL. The V4-V5 region of the 16S rRNA gene was PCR amplified by mixing the following ingredients to a final volume of 25 µL: 12.5 µL of LongAmp^®^ Taq 2x master mix (New England Biolabs, Ipswich, MA, United States ), 1 µL of 10 µM stocks of 515F and 926R primers (Parada et al., 2016) with partial Illumina adapter sequences, 2 µL of DNA sample, and 8.5 µL of nuclease-free water. Samples were amplified according to the following process: denaturation at 94 °C for 30 s, 50 °C for 45 s, and 65 °C for 30 s for 30 cycles, with a final extension at 65 °C for 10 min. Agarose gel electrophoresis was performed to confirm the size and purity of PCR products. PCR products were purified using the DNA Clean and Concentrator kit (Zymo Research, Irvine, CA, United States ) and sent to Genewiz (South Plainfield, NJ, United States ) for Amplicon-EZ Illumina sequencing.

Analysis of demultiplexed and adapter-trimmed reads was performed using USEARCH v11 (Edgar, 2010). Paired-end reads were merged using fastq_mergepairs, length filtered using sortbylength with a minimum sequence length of 400 base pairs, primer sequences were removed using fastx_truncate, and reads were quality filtered using fastq_filter with the fastq_maxee option set to 1.0 to remove reads with greater than one expected error. Unique reads and their abundances were computed using fastx_uniques followed by denoising and chimera removal using the unoise3 command (Edgar, 2016). Reads were mapped to the resulting zero-radius OTUs (ZOTUs) using the otutab command. The taxonomy of the ZOTUs was assigned using the nbc_tax command (Naïve Bayesian Classifier) trained on the RDP 16S database v18 (Cole et al., 2014) with an 80% confidence threshold. ZOTUs below the confidence threshold at a given taxonomic level were labeled as unclassified at that level. Shannon diversity, PERMANOVA, and principal coordinates analysis (PcoA) ordination were computed on the resulting ZOTU tables using scikit-bio’s Diversity, Distance, and Ordination modules, respectively.

## 3 Results

### 3.1 Growth cycle characterization

Consumption of CH_4_ and NH_4_^+^ as well as cell growth were characterized during incubations of low and high resource enrichments to investigate the impact of resource availability on methanotrophic activity.

**FIGURE 1:**
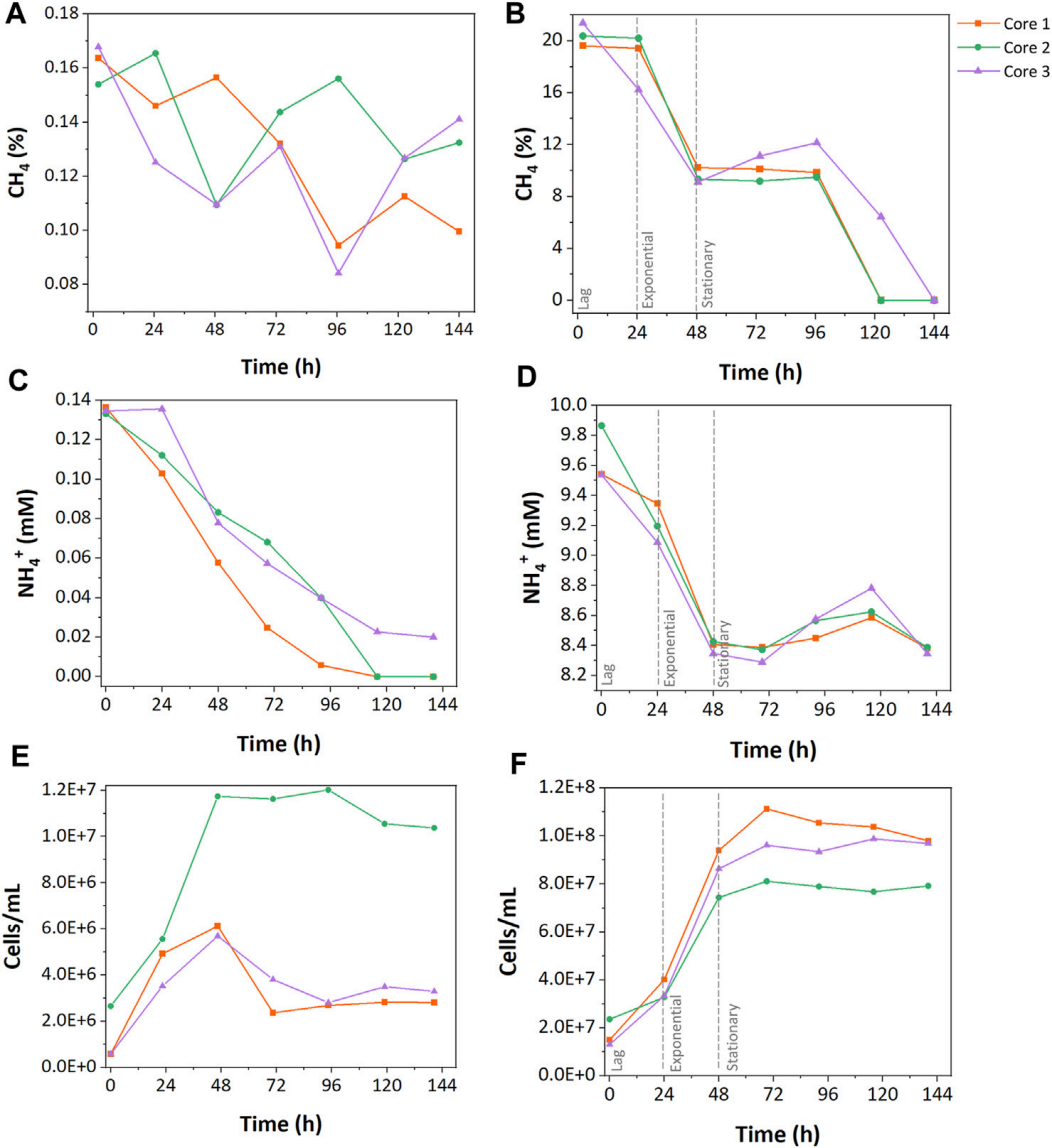
Growth characteristics of low and high resource methanotrophic enrichments from wetlands. CH_4_ consumption **(A,B)**, NH_4_^+^ consumption **(C, D)**, and cell counts **(E,F)**. **(A)**, **(C)**, and **(E)** represent the results of low resource enrichment, and **(B)**, **(D)**, and **(F)** indicate the results of high resource enrichment.

CH_4_ concentrations were monitored to understand how resource concentration affected CH_4_ oxidation. In the low resource enrichments, only 25% ± 10% of the initial CH_4_ was consumed during cultivation with fluctuations. Core 1 consumed 39.2% of the CH_4_, which shows the highest consumption in the low resource enrichments, while core 2 and 3 consumed 20.0% and 15.9% of CH_4_, respectively (Figure 1A). In the high resource enrichments, 53% ± 3.9% of the initially injected CH_4_ was consumed rapidly during the exponential phase followed by the stationary phase (Figure 1B).

NH_4_^+^ was the sole nitrogen source during growth incubations, and its concentration was measured to explore the differences in nitrogen uptake between enrichments. In the low resource enrichments, 85% ± 21% of initially injected NH_4_^+^ was consumed during the growth cycle (Figure 1C). While cores 1 and 2 consumed all the provided NH_4_^+^ during the growth, core 3 only assimilated 55% of the nitrogen source. In contrast, the high resource enrichments consumed only 13% ± 1.3% of total NH_4_^+^ (Figure 1D). No accumulation of NO_2_^−^ or NO_3_^−^ was observed during the incubations suggesting that NH_4_^+^ oxidation was not a major pathway of NH_4_^+^ consumption (Supplementary Table S1).

Cell concentrations of the low and high resource enrichments were measured throughout growth cycles. High resource enrichments contained approximately 10 times more cells compared to the low resource enrichment (high, 7.42 ± 0.975 · 10^7^ cells ∙ mL^-1^; low, 5.91 ± 0.359 · 10^6^ cells ∙ mL^-1^). Additionally, growth curves of the low resource enrichments were more variable between core samples than the high resource enrichments. For instance, low resource enrichment from core 2 grew rapidly up to 1.20 · 10^7^ cells ∙ mL^-1^ and remained stable at that level, while enrichments from cores 1 and 3 only reached 2.75 ± 0.0875 · 10^6^ cells ∙ mL^-1^ and began decaying after 48 h (Figure 1E). In high resource enrichments, a 24 h-lag phase preceded exponential growth within 48 h, followed by a stationary phase (Figure 1F). The maximum cell concentration of the high resource enrichment was 1.11 · 10^8^ cells/mL from the core 1 sample, which was 9.27 times higher than that of the low resource enrichment.

**FIGURE 2:**
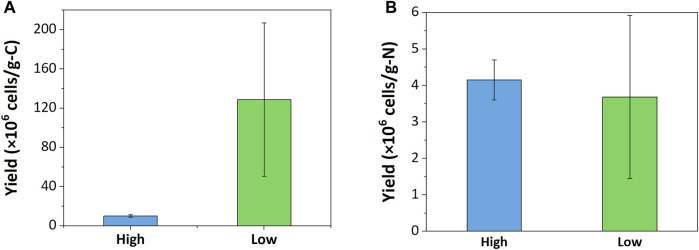
Growth yield based on **(A)** carbon and **(B)** nitrogen consumption.

Carbon- and nitrogen-based cell yields of both enrichments were calculated to evaluate nutrient uptake during growth cycles. The low resource enrichments had 12.7 times higher carbon-based cell yield than the high resource enrichment (Figure 2); the cell yield per consumed CH_4_-C of the low resource enrichment was 128.81 ± 78.24 · 10^6^ cells · g-C^-1^ and that of the high resource enrichment was 10.12 ± 1.39 · 10^6^ cells · g-C^-1^. This aligns well with the observation that a 100-fold increase in carbon supply only increased cell counts by approximately 10-fold. On the other hand, nitrogen-based cell yields of high and low resource enrichments were more comparable; the low resource enrichment had 3.68 ± 2.24 · 10^6^ cells · g-N^-1^ and the high resource enrichment had 4.15 ± 0.55 · 10^6^ cells · g-N^-1^.

### 3.2 Growth limitations in low and high resource enrichments

**FIGURE 3:**
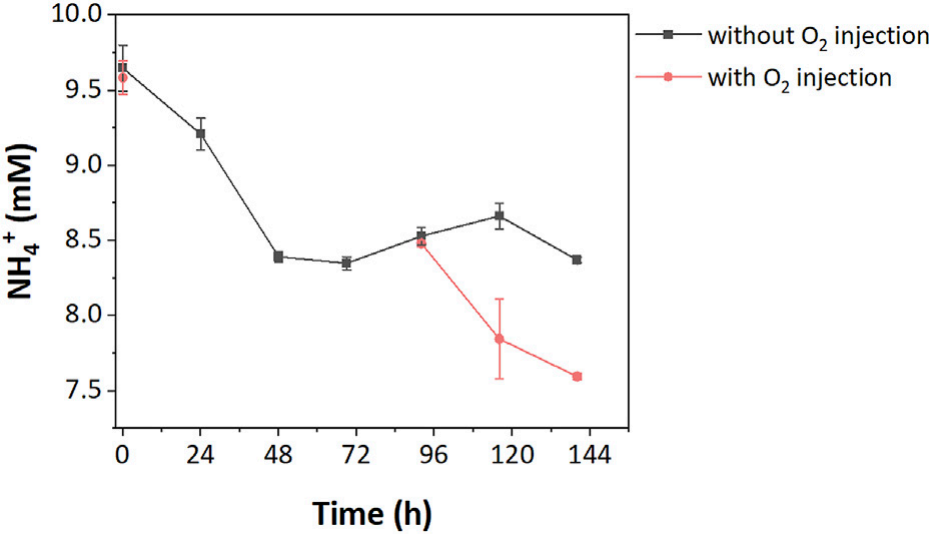
NH_4_^+^ consumption in high resource enrichment with/without O_2_ injection.

Under normal incubation conditions, neither high nor low resource enrichments fully consumed all CH_4_, suggesting other factors were limiting. In the high resource enrichments, we hypothesized oxygen was limiting as a stoichiometric excess of CH_4_ was added during incubations. To test this hypothesis, pure oxygen was injected after the stationary phase (99.5 h). The provision of oxygen led to complete CH_4_ removal, suggesting that oxygen was the rate-limiting factor for CH_4_ oxidation in high resource enrichments. Furthermore, oxygen addition also increased NH_4_^+^ consumption in high resource enrichments with an additional 9.17% of the NH_4_^+^ (i.e., 0.89 mM) being consumed due to growth (Figure 3).

**FIGURE 4:**
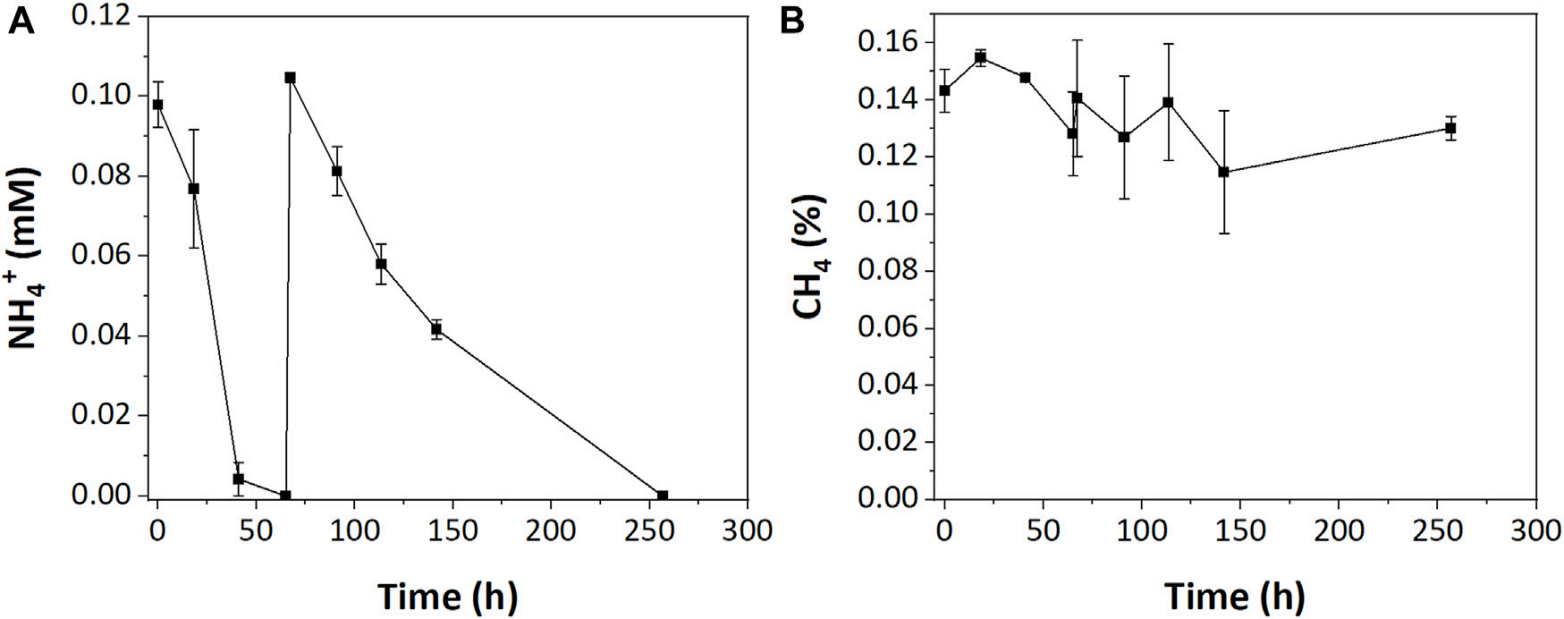
**(A)** NH_4_^+^ and **(B)** CH_4_ consumption in low resource enrichment after additional NH_4_^+^ injection.

In the low resource enrichments, we hypothesized that NH_4_^+^ was limiting due to its complete consumption during incubations. To test this, an additional 0.1 mM of NH_4_^+^ was injected into low resource enrichments after NH_4_^+^ was depleted (67 h). While that additional nitrogen was completely consumed, albeit at a slower rate, it did not stimulate additional CH_4_ oxidation (Figure 4).

### 3.3 PHA accumulation

**FIGURE 5:**
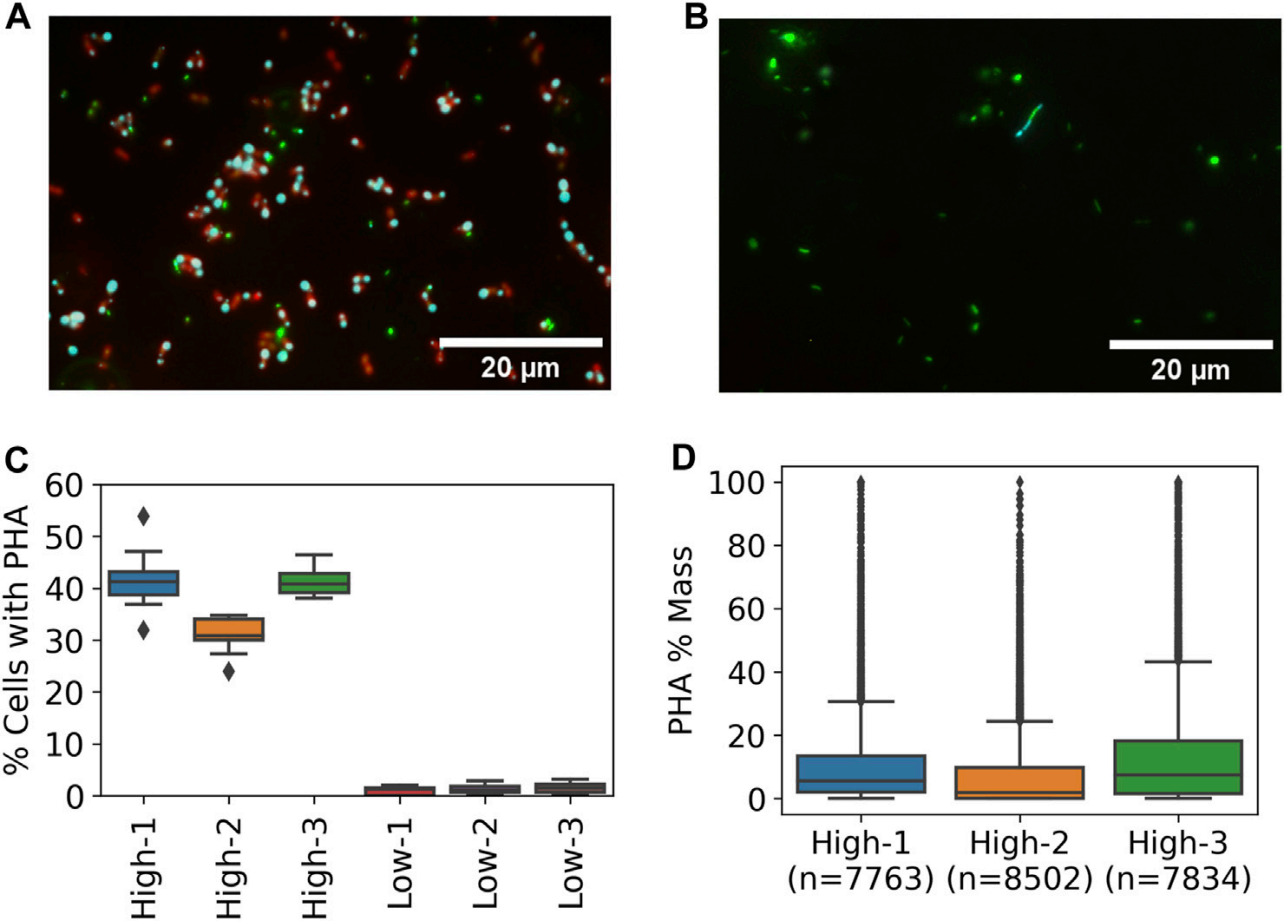
PHA accumulation at high and low resource enrichments based on Nile Blue staining and fluorescence microscopy. Representative epifluorescence images from **(A)** high and **(B)** low enrichments with DNA (green), PHA (cyan), and lipids (red) **(C)** Percent of cells with detectable PHA by microscopic method based on 15 images per sample **(D)** Distribution of single cell PHA content in high resource enrichments based on microscopic method.

The impact of resource availability on PHA-accumulation potential of the enriched methanotrophic communities was tested by running PHA-accumulation cycles and evaluating PHA content by epifluorescence microscopy. The high resource enrichments were dominated by coccoid cells with at least one but mostly two distinct PHA granules present at the poles (Figure 5A). Similar PHA granules were not observed in any of the low resource enrichments though a small fraction of cells exhibited thin bands that stained positive for PHA (Figure 5B).

Quantitative image analysis techniques were used to estimate the frequency of cells containing PHA and the per cell PHA mass fraction based on granule size. An average of 37.9% ± 5.9% of cells in the high resource enrichments contained PHA while only 1.3% ± 0.3% of cells contained PHA signal in the low resource condition (Figure 5C). Furthermore, estimation of PHA content per cell showed that the high resource enrichments accumulated 10.64%, 7.23%, and 14.19% PHA (m/m %) (Figure 5D). These estimates were in good agreement with the results from the traditional GC method which found PHA contents of 13.47%, 9.27%, and 14.95% for the same samples. In addition, each of the three metrics (i.e., percent of cells with PHA, single-cell PHA content, and GC) found the high resource core 2 enrichment to contain less PHA compared to cores 1 and 3.

### 3.4 Community composition and diversity

**FIGURE 6:**
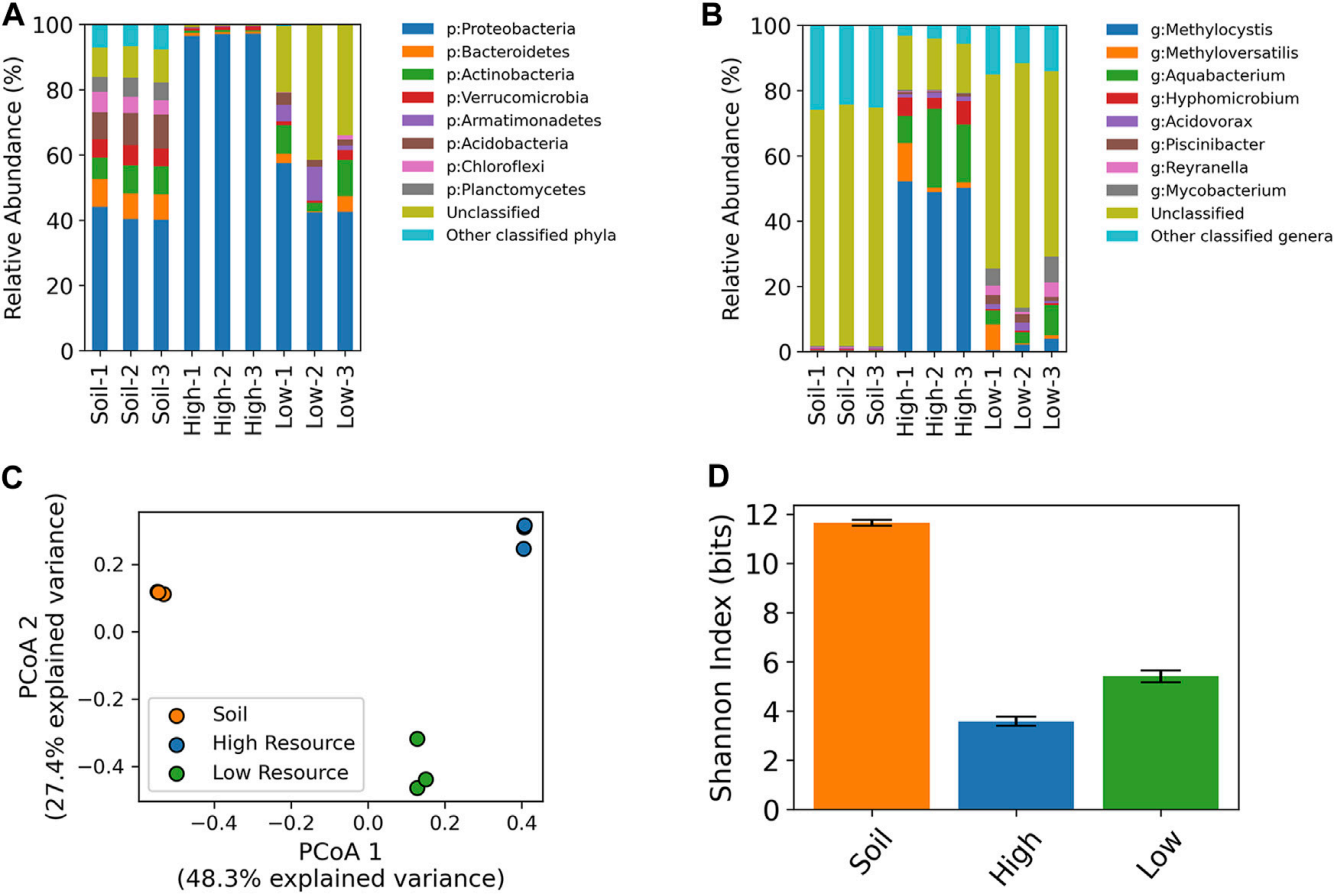
16S rRNA taxonomic profiling of high and low resource enrichment cultures and original wetland soil cores at the phylum **(A)** and genus level **(B)**. Unclassified refers to ZOTUs with a classification confidence of less than 80% at the provided taxonomic level. Principal coordinates analysis of 16S rRNA ZOTU data **(C)**. Shannon diversity index values based on 16S ZOTU tables **(D)**.

Amplicon sequencing of the V4-V5 region of the 16S rRNA gene was conducted on the enrichment cultures and the original wetland soils that were used as inocula. On average, the most abundant phyla in the wetland soil were Proteobacteria (mean ± SD: 41.5% ± 2.2%), Acidobacteria (9.5% ± 1.1%), Bacteroidetes (8.1% ± 0.4%), Actinobacteria (7.9% ± 1.1%), Verrucomicrobia (5.8% ± 0.4%), Planctomycetes (5.3% ± 0.7%), and Chloroflexi (5.2% ± 1.0%) along with a substantial proportion of ZOTUs that were unclassified at the phylum level (9.5% ± 0.6%). The high resource enrichments were dominated by Proteobacteria (96.9% ± 0.4%) with minor populations of Verrucomicrobia (0.9% ± 0.3%), Bacteroidetes (0.8% ± 0.2%), Actinobacteria (0.5% ± 0.2%), while the low resource enrichments consisted of Proteobacteria (47.6% ± 8.6%), Actinobacteria (7.5% ± 4.3%), Armatimonadetes (5.6% ± 4.5%), Bacteroidetes (2.6% ± 2.3%), Acidobacteria (2.6% ± 1.0%), and a large proportion of ZOTUs that were unclassified at the phylum level (31.8% ± 10.7%) (Figure 6A).

At the genus level, the high resource enrichments were dominated by a single *Methylocystis* ZOTU (47.6% ± 1.5%), while the core 1 enrichment had an additional population of *Methyloversatilis* (10.4%). The low resource enrichments harbored these ZOTUs at lower relative abundances with the *Methylocystis* at 2.1% ± 1.7% across cores, while the core 1 enrichment contained the ZOTU classified as *Methyloversatilis* at 7.8%. The starting wetland soil contained *Methylocystis* at 0.2% ± 0.03% and the *Methyloversatilis* ZOTU was only detected in core 1 at 0.001%. (Figure 6B).

Both high and low resource enrichments also contained significant satellite communities. The most abundant classified genus in the low resource enrichments was *Aquabacterium* (5.6% ± 3.1%) followed by *Mycobacterium* (4.8% ± 3.4%). *Aquabacterium* was the second most abundant genera in the high resource enrichments (16.7% ± 8.0%) followed by *Hyphomicrobium* (5.4% ± 2.0%). Closer inspection of the abundant unclassified ZOTUs from the low resource enrichments revealed BLAST hits to uncultured representatives of Planctomycetes (Low-1/ZOTU6), a Vermamoeba mitochondria 16S-like sequence (Low-2/ZOTU3), and an Omnitrophica bacterium (Low-3/ZOTU5).

Beta-diversity analysis (i.e., PCoA) of 16S rRNA gene ZOTU data showed a distinct clustering of the starting wetland soil, high resource enrichments, and low resource enrichments (Figure 6C) (PERMANOVA *p* < 0.01). Moreover, differences in community composition also altered within-community diversity (i.e., alpha diversity). Sample groups had significantly different Shannon indices (One-way ANOVA, p < 1e-7; Tukey’s pairwise HSD, *p* < 0.001) with the wetland soil having the highest, followed by the low resource enrichments, and the high resource enrichments were the least diverse (Figure 6D).

## 4 Discussion

### 4.1 Resource availability shapes methanotrophic and PHA-accumulating functionality

This study investigated the impact of resource concentration on PHA-accumulating methanotrophic enrichments from wetlands. Complete oxidation of supplied CH_4_ was limited in both high and low resource conditions, due to lack of oxygen in the former and presumably due to nitrogen limitation in the latter where the supplied NH_4_^+^ was completely consumed. Though transient nitrogen limitation is known to induce PHA storage, it is not desirable for the selection of communities rich in PHA accumulating organisms. This has been illustrated in acetate (Johnson et al., 2010), fermented molasses (Albuquerque et al., 2007), and CH_4_ (Pieja et al., 2012) fed systems, where nitrogen limitation resulted in a decreasing capacity for PHA storage over time. Such conditions select for faster specific NH_4_^+^ uptake rate and N assimilation which may require use of internal carbon stores and discourage PHA accumulation. As a result, repeated N-limitation likely played a role in the unsuccessful enrichment of PHA-accumulators in the low resource condition.

Low resource concentrations may have also contributed to the lack of PHA accumulators. It has been shown in aerobic granular sludge wastewater treatment systems that alternating conditions of high food-to-microorganisms (F/M) ratios must be applied to out-select the fast-growing heterotrophs and promote the growth of slow-growing PHB accumulating bacteria (de Kreuk et al., 2005; Winkler and Van Loosdrecht, 2022). The high F/M ratio provides PHB storage formers with the competitive edge to acquire more substrate through non-growth associated fast substrate uptake (q_s, max_) that is multiple times faster than substrate usage through canonical growth (µ_max_) without storage (Winkler et al., 2015; Chen et al., 2020). In our low resource condition, the F/M ratio may not have been high enough to select for PHA storage formers while in the high resource condition it was sufficient.

We observed a much higher carbon-based cell yield in the low resource condition, while the cell yield per N remained similar across conditions; a similar observation was made in nitrogen-limited acetate-fed cultures during the feast phase of growth (Johnson et al., 2010). The differences in yield may be attributed to C uptake and storage as PHA in the high resource condition leading to a decreased carbon use efficiency compared to the low condition where a higher proportion of available carbon was directed towards growth. Cross-feeding and resource exchange in the low resource condition could have also contributed to greater carbon use efficiency.

In line with the literature showing that repeated nitrogen limitation and low F/M ratios will not favor PHA accumulation, our results showed that the low resource enrichments harbored little PHA storage capacity compared to the high resource enrichments (1.3% and 37.9% of cells containing PHA respectively). This difference in PHA storage was comparable to the difference in the relative abundance of *Methylocystis* methanotrophs in the two conditions (2.1% and 47.6% in the low and high resource enrichments respectively). Microscopic imaging of the cultures after incubation in nitrogen-free media provided a clear view of the distribution of PHA-harboring microorganisms in the community and showed the location of PHA granules within the cell. Furthermore, quantitative image processing showed good agreement with the traditional GC method of PHA quantification. We extended the Nile Blue A staining method from Lazic *et al.* by including a SYBR Green stain for nucleic acids and used epifluorescence microscopy as opposed to confocal which requires more advanced instrumentation. Utilization of the SYBR Green stain revealed that the Nile Blue did not produce signal for the membranes of all cells especially in the low resource enrichments (Supplementary Figure S1), which could be due to differences in membrane composition of different organisms.

The PHA content of the high resource enrichments (12.6% ± 2.4%) was lower than other studies investigating methanotrophs as PHA producers, however, if normalized by the relative abundance of methanotrophs, then the PHA content is more in line with literature reports (26.4% ± 6.1%). For example, others have reported 22.2% PHA from a rice field soil enrichment (Kulkarni et al., 2022), 39% from an activated sludge enrichment (Myung et al., 2015), 43.1% from a pure culture *Methylocystis hirsuta* fed with biogas (López et al., 2018), and 25.9% from a pure culture of *Methylocystis* sp. Rockwell in nitrogen-free media (Lazic et al., 2022). The oxygen limitation experienced by our high resource enrichments could have contributed to the limited PHA production as this has been shown to negatively affect methanotroph PHA production (Zaldívar Carrillo et al., 2018).

### 4.2 Resource availability shapes community structure

Despite these differences in growth characteristics, the high- and low resource enrichments contained the same *Methylocystis* ZOTU, albeit at different abundances and surrounded by different satellite communities. Methanotrophs from the genus *Methylocystis* have been enriched from a variety of environments including wetlands (Wartiainen et al., 2006), rice fields (Kulkarni et al., 2022) and activated sludge (Myung et al., 2015). In addition, the *Methylocystis* genus has been studied extensively for the production of PHA (Pieja et al., 2011; Lazic et al., 2022), so the enrichment of a *Methylocystis* type methanotroph in this study was not surprising. Our high resource enrichments contained only about 47.6% methanotrophs while others have reached 65.0%–77.2% (Myung et al., 2015) and 88.92% (Kulkarni et al., 2022), which may provide additional context to the lower overall PHA content observed in this report. Interestingly, the low resource enrichments also harbored a population of *Mycobacterium*, a genus in which a methanotrophic organism was recently identified (van Spanning et al., 2022), though most *Mycobacterium* are not methanotrophic.

Low resource enrichments were significantly more diverse than high resource enrichments, which is in alignment with bioreactor systems operated at high organic carbon concentrations that are enriched with just a few major ZOTUs (Sato et al., 2016). The relationship between nutrient concentration and microbial diversity has been explored in aquatic ecosystems (Smith, 2007), soils (Bastida et al., 2021), a photosynthetic mat (Bernstein et al., 2017), and laboratory serial dilution culture systems (Erez et al., 2020) with varying results. The latter describes an effect where large nutrient additions favor a subset of “early-bird” species that undergo rapid growth to numerically dominate the system while smaller nutrient additions are analogous to a chemostat-like system where metabolic trade-offs enable an arbitrarily large number of species to coexist (Erez et al., 2020). Results from our enrichments support this idea that high nutrient concentrations allowed for the dominance of a single “early-bird” ZOTU while the low nutrient concentrations created a more diverse and even community. The increased diversity in the low resource enrichments may have also contributed to the variability in growth characteristics, particularly in cell concentrations, compared to the high resource enrichments that harbored a dominant population of methanotrophs.

### 4.3 Implications for biorefinery applications

This study provides a few takeaways useful for the development of CH_4_-fed biorefineries. First, we were unable to select for a significant population of PHA-accumulating methanotrophs under the low resource condition, which highlights the challenge of using dilute CH_4_ sources like landfills, cattle operations, or ventilated coal mines. Future work should address these issues, since dilute sources comprise a significant proportion of anthropogenic CH_4_ emissions (Saunois et al., 2020). In the case of concentrated CH_4_ streams, we found that enrichments consumed only a fraction of the supplied NH_4_^+^, suggesting that it may be possible to cultivate PHA-accumulating biomass at higher C/N ratios by reducing N-supply. This could reduce operational costs of the system and highlight the importance of tracking nitrogen consumption. Finally, we show that PHA-accumulating methanotrophs can be sourced from freshwater wetlands, which could serve as a promising inoculum for CH_4_-fed biorefineries.

### 4.4 Implications for natural ecosystems

The subject of microbial storage has been reviewed in the context of soil ecosystems where it is a widespread trait that aids in effective resource allocation and survival and plays a role in the soil carbon cycle (Mason-Jones et al., 2022). In wetlands, the conversion of CH_4_ into PHA by methanotrophs could enhance carbon storage and decrease emissions from the system. This study shows that PHA-accumulating methanotrophs can be enriched from wetland soil in high resource condition. Though we did not observe PHA in the low resource condition meant to mimic the natural system, field studies have measured gas bubbles from wetlands with CH_4_ concentrations in the range of 50%–80% (Chanton et al., 1989), so the high resource condition still holds some relevance to the natural system. It is possible that rapid carbon uptake and storage by methanotrophs occurs under transient exposure to CH_4_-rich bubbles as they leave the wetland or form on surfaces. Future studies exploring this phenomenon could further illuminate the environmental relevance of methanotrophic PHA storage in wetland soils.

## 5 Conclusion

Methanotrophic communities dominated by *Methylocystis*-associated organisms were enriched from freshwater wetland soils under high and low resource concentrations at a fixed C/N ratio. The low nutrient enrichments became nitrogen-limited and did not select for PHA accumulation but retained a higher level of diversity based on 16S rRNA amplicon sequencing. In contrast, the high nutrient enrichments became oxygen limited, contained a sizable population of PHA-accumulating organisms, and had a lower level of diversity. The Nile Blue and SYBR Green fluorescent staining method enabled visualization and quantification of PHA in the mixed communities and showed good agreement with the traditional GC method. Overall, this study highlights the importance of resource concentration as a factor on top of C/N-ratios when selecting for PHA accumulating methanotrophic communities and points to their relevance in natural wetland systems.

## Data Availability

Raw sequencing data from this study is available in the NCBI Sequence Read Archive (SRA) under Bioproject PRJNA954053 and Biosamples SAMN34133114, SAMN34133115, SAMN34133116, SAMN34133117, SAMN34133118, SAMN34133119, SAMN34133120, SAMN34133121, and SAMN34133122. Sequencing and image analysis code can be found in a GitHub repository for this project (https://github.com/zflink/Wetland_Methanotrophs_PHA).
