# Supplementary material for: Resource availability governs polyhydroxyalkanoate (PHA) accumulation and diversity of methanotrophic enrichments from wetlands
Source: Front Bioeng Biotechnol. 2023 Jul 31;11:1210392. doi: 10.3389/fbioe.2023.1210392 (PMC10425282; doi:10.3389/fbioe.2023.1210392)
Supplement: Supplementary file 1 [file DataSheet1.docx]

Supplementary Material

Resource availability governs polyhydroxyalkanoate (PHA) accumulation and diversity of methanotrophic enrichments from wetlands

Yujin Kim†, Zachary Flinkstrom†, Pieter Candry*, Mari Winkler, Jaewook Myung*

† These authors contributed equally to this work and share the first authorship.

*** Correspondence:** Pieter Candry: pcantry@uw.edu, Jaewook Myung: jjaimyung@kaist.ac.kr

# Supplementary Figures and Tables


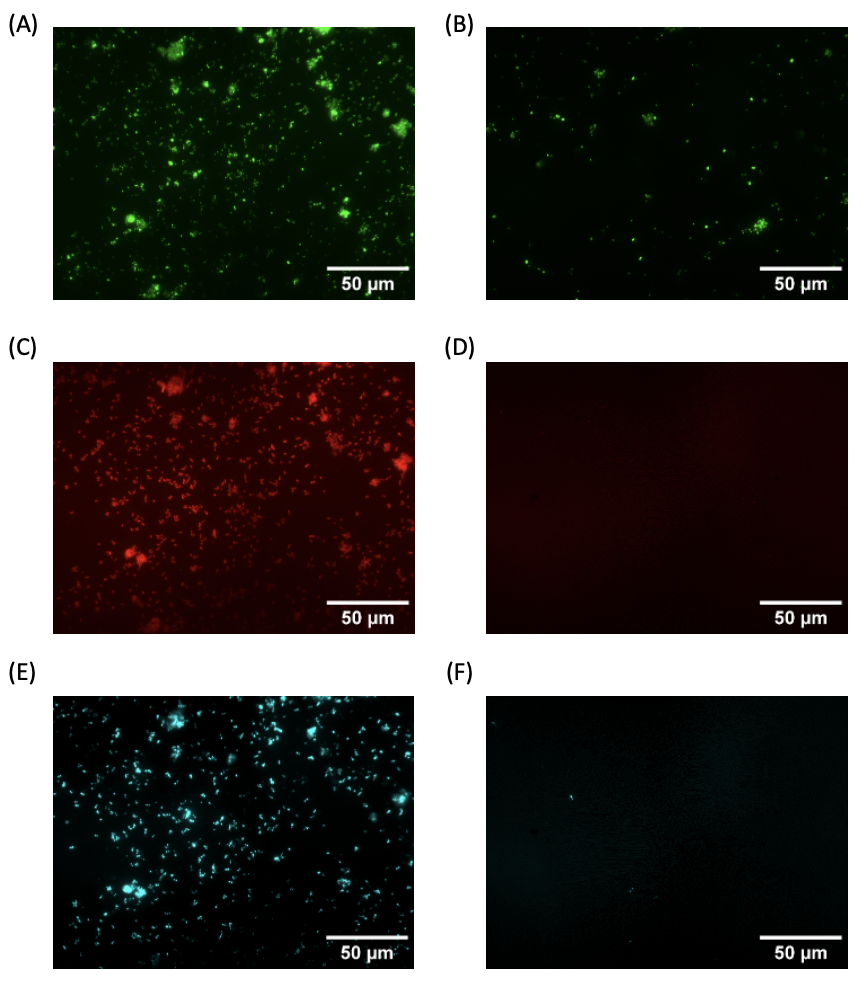


**Supplementary Figure 1.** Epifluorescence microscopic images showing staining in different channels for high (A, C, E) and low (B, D, F) resource enrichments. Sybr Green DNA staining is shown in green (A, B), Nile Blue lipid staining is shown in red (C, D), and Nile Blue PHA staining is shown in cyan (E, F).

**
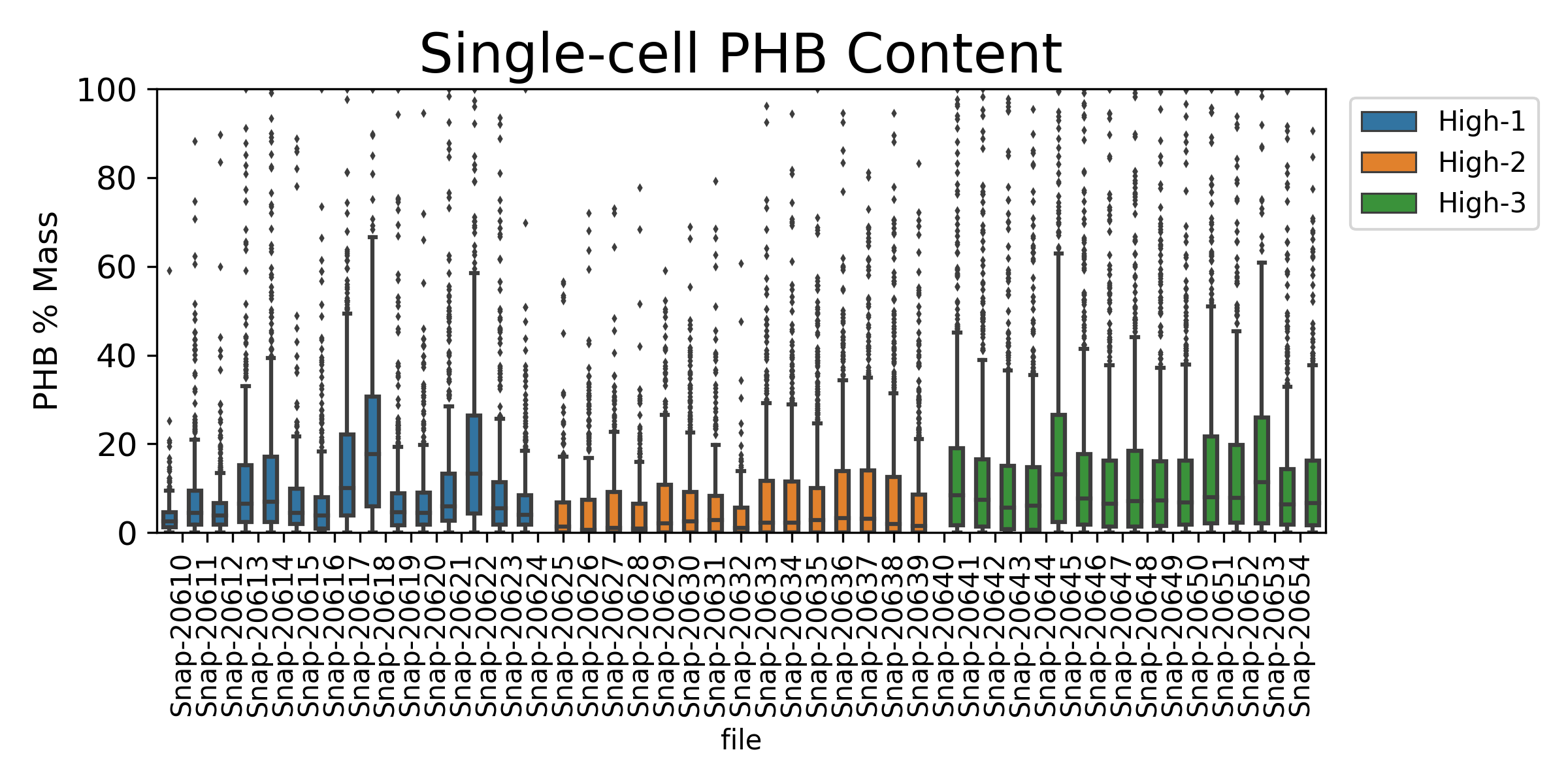
**

**Supplementary Figure 2.** Single-cell PHB % (m/m) estimates from Nile Blue staining method separated by each image analyzed (n=15 per enrichment). Figure 5d in the main text represents the aggregate of the images for each enrichment replicate (i.e., High-1, High-2, and High-3).


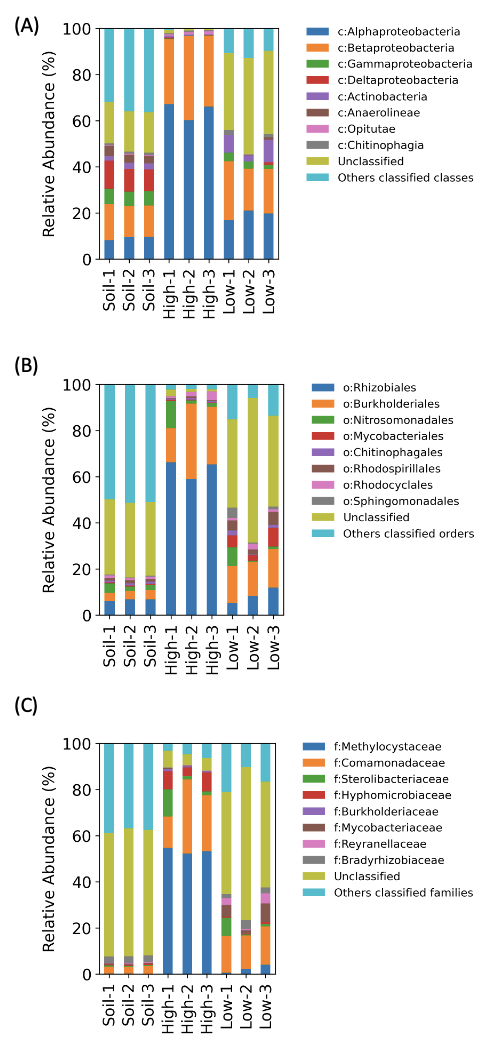


**Supplementary Figure 3.** Taxonomic composition of samples based on 16S rRNA amplicon sequencing at the class (A), order (B), and family (C) level. Unclassified refers to ZOTUs with a classification confidence of less than 80% at the provided taxonomic level.

| **High resource enrichments** | | | | | | | |
| --- | --- | --- | --- | --- | --- | --- | --- |
| **Time (h)** | **0** | **24** | **48** | **69** | **92** | **116** | **140** |
| **NH_4_^+^ (mM)** | 9.65 | 9.21 | 8.39 | 8.35 | 8.53 | 8.66 | 8.37 |
| **(std)** | (0.154) | (0.106) | (0.034) | (0.043) | (0.058) | (0.085) | (0.019) |
| **NO_2_^-^ (mM)** | BLoD | BLoD | BLoD | BLoD | BLoD | BLoD | BLoD |
| **TON (mM)** | BLoD | BLoD | BLoD | BLoD | BLoD | BLoD | BLoD |
| **Low resource enrichments** | | | | | | | |
| **Time (h)** | **0** | **24** | **48** | **69** | **92** | **116** | **140** |
| **NH_4_^+^ (mM)** | 0.135 | 0.117 | 0.078 | 0.057 | 0.040 | 0.023 | 0.020 |
| **(std)** | (0.0013) | (0.0138) | (0.0148) | (0.0234) | (0.0276) | (0.0321) | (0.0283) |
| **NO_2_^-^ (mM)** | BLoD | BLoD | BLoD | BLoD | BLoD | BLoD | BLoD |
| **TON (mM)** | BLoD | BLoD | BLoD | BLoD | BLoD | BLoD | BLoD |

**Supplementary Table 1.** Mean concentrations of NH_4_^+^, NO_2_^-^, and TON (NO_3_^-^ + NO_2_^-^) (mM) during the incubation of high and low resource enrichments without additional oxygen addition. BLoD – Below Limit of Detection.
